# Asymptomatic Bochdalek's Hernia in an Adult: A Case Report

**DOI:** 10.7759/cureus.59635

**Published:** 2024-05-04

**Authors:** Petr Lochman, Michal Hůlek, Tomáš Dušek

**Affiliations:** 1 Department of Military Surgery, University of Defence, Military Faculty of Medicine, Hradec Kralove, CZE; 2 Department of Surgery, University Hospital, Hradec Kralove, CZE; 3 Department of Radiology, University Hospital, Hradec Kralove, CZE

**Keywords:** bochdalek's hernia, asymptomatic, surgical treatment, adulthood, congenital diaphragmatic hernia

## Abstract

Bochdalek's hernia is the most common congenital malformation of the diaphragm with a defect in its posterolateral part. Its clinical manifestation in adulthood is rare. It is often an incidental finding, and its diagnosis may be challenging. A high index of suspicion is necessary, especially in cases presenting with cardiopulmonary or abdominal symptoms and an ambiguous finding on the initial chest X-ray.

We present a case of an asymptomatic 50-year-old male patient with a bulky left-sided Bochdalek's hernia. Surgical treatment was indicated, and a direct suture of the defect after reduction of the herniated greater omentum, transverse colon, and tail of the pancreas was performed from the upper midline laparotomy. The postoperative course was uneventful, and the patient was discharged on the fifth postoperative day. The management of adult patients with these kinds of hernias in both acute and chronic settings is discussed, and some recommendations are mentioned to minimize unnecessary pitfalls.

## Introduction

Congenital diaphragmatic hernias (CDH) occur with an incidence of one in 2,000-3,000 newborns and are mostly diagnosed and treated in early childhood [1, 2]. Bochdalek's hernia is the most common type of CDH, which consists of a defect in the posterolateral aspect of the diaphragm, predominantly on the left side (75%-90%) [3, 4]. It was first described by the Czech anatomist Vincent Alexander Bochdalek in 1848 [1].

Bochdalek's hernia is mostly diagnosed and treated in early childhood. Its manifestation in adults is quite rare, with an incidence of about 0.17%, and only a few tens of cases have been reported so far [3, 5-10]. We present a case report of a completely asymptomatic 50-year-old man with a giant left-sided Bochdalek's hernia that was an incidental finding on a chest X-ray. The patient was indicated for an elective operation, and closure of the defect from the upper midline laparotomy was performed. The management of adult patients with Bochdalek's hernia, both symptomatic and asymptomatic, is further discussed, and some tips to avoid potential pitfalls are emphasized.

## Case presentation

**Figure 1:**
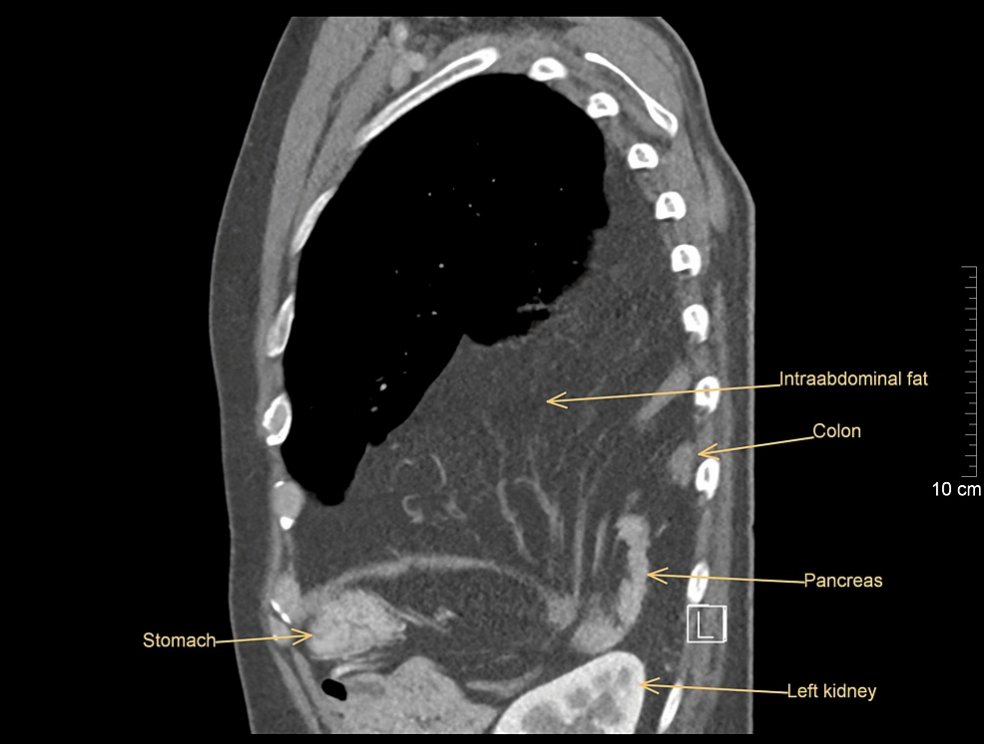
Herniated omentum, transverse colon, and tail of the pancreas (sagittal view)

**Figure 2:**
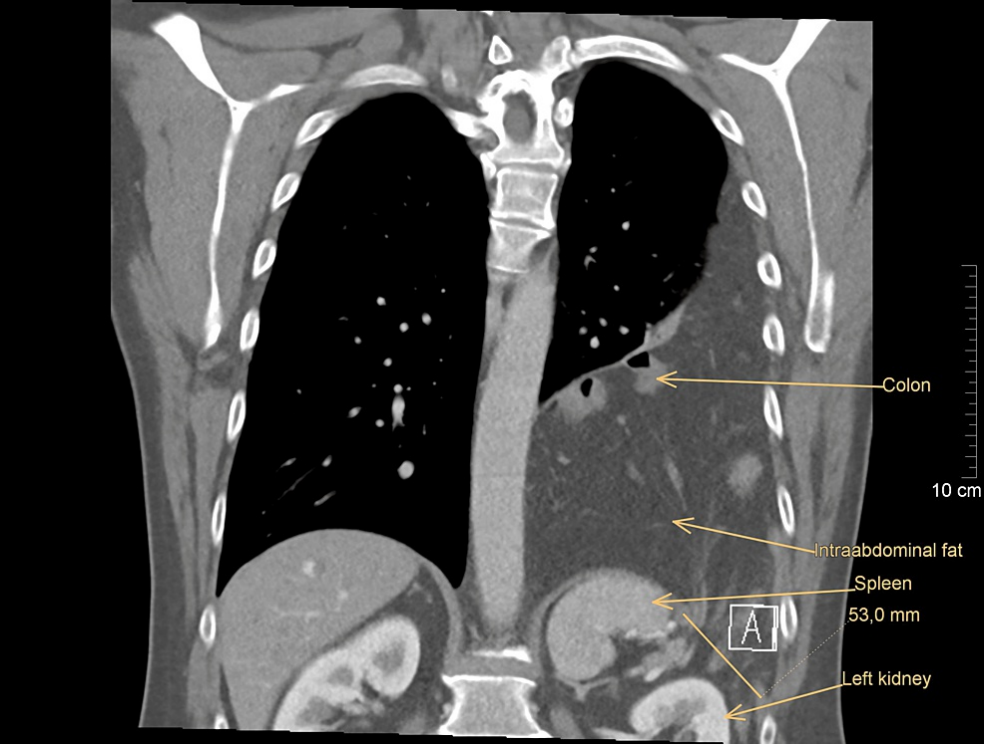
Left-sided Bochdalek's hernia with a 53-mm defect lateral to the spleen (frontal view)

A 50-year-old Caucasian male presented to the outpatient department of the pulmonary clinic with an incidental finding of shadowing of the left lung on a chest X-ray performed as part of a regular medical check-up. He was completely asymptomatic, with no associated comorbidities, no history of previous trauma or surgery, and no negative family history. He had been a chain smoker for about 30 years (30 cigarettes per day) and worked as a heating engineer with short-term contact with asbestos in the past. To rule out a lung tumor, a whole-body contrast-enhanced CT was indicated and revealed a bulky left-sided Bochdalek's hernia. The round defect in the posterolateral part of the left hemidiaphragm was 53 mm large and had a greater omentum; the major part of the transverse colon and the tail of the pancreas were displaced into the left pleural cavity (Figures 1, 2). The subsequent functional pulmonary examination showed only a mild restrictive ventilation disorder; the blood tests were within the normal range.

The patient was indicated for surgical treatment. Reduction of the herniated organs back into the abdominal cavity was performed from the upper midline laparotomy, and the 8x4 cm round defect in the diaphragm was closed with an interrupted absorbable suture in one layer. The postoperative course was uneventful, and the patient was discharged to outpatient care on the fifth postoperative day.

## Discussion

The clinical manifestation of Bochdalek's hernia in adulthood is heterogeneous, ranging from completely asymptomatic cases to chronic, non-specific problems to abdominal emergencies. The most common symptoms are respiratory (dyspnea, cough, possibly circulatory instability) and gastrointestinal (abdominal pain, intestinal obstruction, or signs of perforation) [11, 12]. Possible predisposing factors leading to symptoms in adults include conditions with increased intra-abdominal pressure (pregnancy, chronic constipation, blunt abdominal trauma), multiple laparoscopies in a short period of time, or a persistent cough [12].

Diagnosis may not be easy, even in symptomatic cases, and it is often an incidental finding. The first examination is usually a chest X-ray, where, especially in the case of unclear shadowing or the presence of an air bubble in the pleural cavity, there should be a high level of suspicion for this diagnosis. A thoracic ultrasound can lead to more precise findings, which can also prevent complications during a chest puncture or the introduction of a chest tube [9]. The method of choice is a contrast-enhanced CT of the chest and abdomen, which confirms the diagnosis and describes the herniated content and size of the defect [1]. The sensitivity of the examination is 78% for the left-sided hernia and 50% for the right one [3]. The CT findings can also contribute to the decision on the choice of surgical approach when complications such as hollow viscera perforation or mechanical bowel obstruction are diagnosed.

There is a consensus on the appropriateness of the operative treatment, but relevant guidelines are still lacking [2, 12], although some recommendations for the management of complicated diaphragmatic hernia in the acute setting were published [13]. The surgical approach is mainly determined by the acuteness of the condition, the general condition of the patient, the lateralization and size of the finding, and the experience of an individual surgeon (general or thoracic), of course [1, 3, 11, 12]. In general, a laparotomy or thoracotomy can be used, but in cases of minor defects, especially in asymptomatic patients, a mini-invasive approach (laparoscopy or thoracoscopy) is preferred. Recently, cases managed by a robotic approach have also been published. All approaches have their pros and cons; in some cases, a combination of both can be useful or inevitable. During laparotomy (laparoscopy), the advantage is the inspection of reduced herniated organs into the abdominal cavity with the possibility of their treatment after reduction, while during thoracotomy (thoracoscopy), direct visual control of adhesiolysis is often necessary, especially in long-lasting conditions. A transthoracic approach is advantageous when the hernia is located on the right side, as well. An open approach is generally preferred in acute settings [9, 13].

The method of closure of the defect consists of a simple suture, usually with interrupted non-absorbable stitches in one or two layers, or the use of prosthetic or biological mesh for larger defects when a direct suture without tension is not possible. The reinforcement of the direct suture by applying mesh remains questionable [11].

Immediate postoperative complications usually correspond to the chosen surgical approach (respiratory or abdominal) and are managed conservatively in the majority of cases [3, 11, 12]. Data on long-term results are lacking, with exceptions. However, a low recurrence rate prevails in published cases with a long postoperative follow-up [12].

## Conclusions

Bochdalek's hernia is the most common congenital malformation of the diaphragm, with a rare manifestation in adulthood. It is often an incidental finding, and its diagnosis may be challenging due to vague symptoms. Even in asymptomatic patients, like in our presented case, surgical treatment is highly recommended to prevent potential complications in the future. The mini-invasive approach (laparoscopy or thoracoscopy) with direct sutures of smaller defects or using an implant has been gradually increasing in chronic settings, while open laparotomy or thoracotomy prevails in acute cases even though the guidelines are still missing. 
